# Investigating the Role of Functional Polymorphism of Maternal and Neonatal Vitamin D Binding Protein in the Context of 25-Hydroxyvitamin D Cutoffs as Determinants of Maternal-Neonatal Vitamin D Status Profiles in a Sunny Mediterranean Region

**DOI:** 10.3390/nu13093082

**Published:** 2021-09-01

**Authors:** Spyridon N. Karras, Erdinç Dursun, Merve Alaylıoğlu, Duygu Gezen-Ak, Cedric Annweiler, Fatme Al Anouti, Hana M. A. Fakhoury, Alkiviadis Bais, Dimitrios Kiortsis

**Affiliations:** 1 National Scholarship Foundation, 55535 Thessaloniki, Greece; 2 Brain and Neurodegenerative Disorders Research Laboratories, Department of Medical Biology, Cerrahpasa Faculty of Medicine, Istanbul University-Cerrahpasa, 34381 Istanbul, Turkey; erdincdu@gmail.com (E.D.); merve.alaylioglu@hotmail.com (M.A.); duygugezenak@gmail.com (D.G.-A.); 3 Department of Neuroscience, Institute of Neurological Sciences, Istanbul University-Cerrahpasa, 34381 Istanbul, Turkey; 4 Department of Geriatric Medicine and Memory Clinic, Research Center on Autonomy and Longevity, University Hospital, 49035 Angers, France; CeAnnweiler@chu-angers.fr; 5 Department of Medical Biophysics, Robarts Research Institute, Schulich School of Medicine and Dentistry, The University of Western Ontario, London, ON N6A 3K7, Canada; 6 Department of Health Sciences, College of Natural and Health Sciences, Zayed University, Abu Dhabi 144534, United Arab Emirates; Fatme.AlAnouti@zu.ac.ae; 7 Department of Biochemistry and Molecular Biology, College of Medicine, AlFaisal University, Riyadh 11533, Saudi Arabia; hana.fakhoury@gmail.com; 8 Laboratory of Atmospheric Physics, Aristotle University of Thessaloniki, 54124 Thessaloniki, Greece; abais@auth.gr; 9 Department of Nuclear Medicine, University of Ioannina, 45110 Ioannina, Greece; dkiorts@uoi.gr

**Keywords:** vitamin D, pregnancy, neonatal health, functional polymorphism

## Abstract

Recent results indicate that dysregulation of vitamin D-binding protein (VDBP) could be involved in the development of hypovitaminosis D, and it comprises a risk factor for adverse fetal, maternal and neonatal outcomes. Until recently, there was a paucity of results regarding the effect of maternal and neonatal VDBP polymorphisms on vitamin D status during pregnancy in the Mediterranean region, with a high prevalence of hypovitaminosis D. We aimed to evaluate the combined effect of maternal and neonatal VDBP polymorphisms and different maternal and neonatal 25-hydroxyvitamin D (25(OH)D) cut-offs on maternal and neonatal vitamin D profile. Blood samples were obtained from a cohort of 66 mother–child pairs at birth. Our results revealed that: (i) Maternal VDBP polymorphisms do not affect neonatal vitamin D status at birth, in any given internationally adopted maternal or neonatal cut-off for 25(OH)D concentrations; (ii) neonatal VDBP polymorphisms are not implicated in the regulation of neonatal vitamin D status at birth; (iii) comparing the distributions of maternal VDBP polymorphisms and maternal 25(OH)D concentrations, with cut-offs at birth, revealed that mothers with a CC genotype for rs2298850 and a CC genotype for rs4588 tended to demonstrate higher 25(OH)D (≥75 nmol/L) during delivery (*p* = 0.05 and *p* = 0.04, respectively), after adjustments for biofactors that affect vitamin D equilibrium, including UVB, BMI and weeks of gestation. In conclusion, this study from Southern Europe indicates that maternal and neonatal VDBP polymorphisms do not affect neonatal vitamin D status at birth, whereas mothers with CC genotype for rs2298850 and CC genotype for rs4588 demonstrate higher 25(OH)D concentrations. Future larger studies are required to establish a causative effect of these specific polymorphisms in the attainment of an adequate (≥75 nmol/L) maternal vitamin D status during pregnancy.

## 1. Introduction

Vitamin D has gained a tremendous width of ongoing scientific research during the past two decades [1,2,3]. Its undisputed role in bone mineralization has been expanded to a widely adopted hypothesis, associating maternal hypovitaminosis D during pregnancy with an increased risk of the development of adverse pregnancy outcomes and impairment of future offspring’s metabolic health [4,5]. Mechanistic evidence reported that maternal 25-hydroxyvitamin D (25(OH)D) correlates strongly with neonatal 25(OH)D concentrations at birth [6,7,8]. On the other hand, there is a continuing scientific debate and differing criteria of maternal and neonatal vitamin D deficiency worldwide [9,10]. The main reasons for this wide controversy might implicate individual genetic and regional characteristics [11,12], including ethnic variations of vitamin D receptor (VDR) polymorphisms [13], ultraviolet B (UVB) radiation [14] and country-specific dietary patterns [15]. The potential influence of the specific genetic background of each individual for decreasing pregnancy complications and optimizing neonatal health could provide a holistic and personalized clinical approach in daily practice and future vitamin D supplementation practices during pregnancy.

Vitamin D binding protein (VDBP) comprises one of the most important factors for vitamin D metabolism [16,17,18,19]. Previous results outline that VDBP metabolism disorders comprise a risk factor for adverse maternal and neonatal outcomes [20,21,22,23,24]. These reports vary according to regional and population parameters, including most European countries, with different public health strategies.

The prevailing view for most eastern European and Mediterranean pregnant populations has been, for decades, that casual exposure to sunlight provides enough vitamin D. Observational data from this region reported a high prevalence of vitamin D deficiency during pregnancy [25,26]. Until recently, there was a paucity of results regarding the effect of maternal and neonatal VDBP polymorphisms on vitamin D metabolism during pregnancy within this region. In addition, so far a combined clinical (in terms of various maternal/neonatal 25(OH) D cut-offs) and genetic (including different maternal and neonatal VDBP polymorphisms) approach has not been investigated.

We aimed to evaluate the combined effect of maternal and neonatal VDBP polymorphisms and different maternal and neonatal 25(OH) D cut-offs on the profiles of maternal and neonatal vitamin D status, within a sunny region of the Mediterranean basin.

## 2. Methods

### 2.1. Inclusion and Exclusion Criteria

The study included a cohort of mother–child pairs at birth. Inclusion and exclusion criteria have been previously described [7,13]. Use of vitamin D supplements was also an exclusion criterion. Daily calcium (Ca) supplement use was also recorded. Informed consent was obtained. The study was conducted from January 2018 to September 2018. The study was approved by the Bioethics Committee of the Aristotle University of Thessaloniki, Greece (approval number 1/19-12-2011).

### 2.2. Demographic and Dietary Data—Biochemical and Hormonal Assays

At enrollment, demographic and social characteristics were recorded. Ca and vitamin D dietary intake during the last month of pregnancy were assessed through a validated semi-quantitative food frequency questionnaire that includes 150 food and beverages [27]. From these data, calculations were made for estimations of consumed quantities (in g per day) based on a food composition database, based on the Greek diet [27] for estimating daily dietary Ca and vitamin D intake. Maternal alcohol use during pregnancy was defined either as none (subdivided into never drinking alcohol or drinking alcohol but not during pregnancy), light (1–2 units per week or at any one time during pregnancy) or moderate (3–6 units per week or at any one time during pregnancy) [28].

Blood samples were obtained from mothers 30–60 min before delivery. Umbilical cord blood was collected, immediately after clamping, from the umbilical vein. Concentrations of 25(OH)D2 and 25(OH)D were determined using liquid chromatography–tandem mass spectrometry (LC–MS/MS), with lower limits of quantification (LLOQ); 25(OH)D2 (0.5 ng/mL), 25(OH)D (0.5 ng/mL) and the sum of both vitamin D forms is provided as total 25 (OH)D [29,30].

### 2.3. Neonatal and Maternal Vitamin D Status Cut-Offs and Combined VDBP Polymorphisms Evaluation

Differences in the frequency of vitamin D status according to neonatal and maternal VDBP polymorphisms were determined according to their vitamin D status at birth: 25(OH)D ≤ 25 nmol/L (deficiency), 25–50 nmol/L (insufficiency) and 25(OH)D ≥ 50 nmol/L (sufficiency) [2,4,5,6]. Following this classification, maternal and neonatal VDBP polymorphisms were assessed at birth to investigate potential differences of maternal and neonatal vitamin D status.

### 2.4. VDBP Analysis

DNA isolation was performed by QIAamp DNA Blood Mini Kit (Cat. No. 51304, QIAGEN, Hilden, Germany) according to the manufacturer’s protocol. Genotypes of VDBP rs2298850, rs4588 and rs7041 SNPs were determined by LightSNiP assay using simple probes (LightSNiP, TibMolBiol, Berlin, Germany) and LightCycler Fast Start DNA Master HybProbe Kit (Cat. No.12239272001, Roche Diagnostics, Mannheim, Germany). Real-time PCR (RT-PCR) was performed with LightCycler 480 Instrument II (Roche Diagnostics, Mannheim, Germany), and genotyping was done by using melting curve analysis as previously described [31].

### 2.5. UVB Measurements

UVB data for the broad geographical region of Thessaloniki, Greece, were collected at the Laboratory of Atmospheric Physics, School of Physics, Aristotle University of Thessaloniki. The daily integral of vitamin D effective UVB radiation (09:00 to16:00 local time) was expressed as the amount of sunlight hitting a horizontal surface, updated every five minutes, in watts per hour square meter (wh/m^2^). Mean UVB exposure during the previous 45 days (daily integral) before blood sample collection (estimated mean half-life of vitamin D) was calculated.

### 2.6. Statistical Analysis

All analyses that involve the distributions of genotypes of VDBP polymorphisms were analyzed with the chi-square (χ^2^) test, df:2 for genotypes. Significance was also confirmed with Cramer’s V/Kendall’s tau-c. The comparisons between mean values of the groups were performed with one-way ANOVA followed by multiple comparison tests, either Tukey HSD or Dunett C, depending on the normality of the data set. Homogeneity of variances was checked with Levene’s Test for homogeneity of variances. When required, the data and *p* values were adjusted for maternal height (cm), BMI pre-pregnancy (kg/m^2^), BMI terminal (kg/m^2^), UVB and weeks of gestation by one-way analysis of covariance (ANCOVA). All data are presented as the mean ± SD in the text and figure legends. *p* values lower than 0.05 were considered statistically significant. “SPSS 24.0” software was used in these comparisons.

## 3. Results

Seventy mother–neonate dyads were initially included. Given four neonates had missing birth biochemical data, they were excluded from the related analysis. Demographic, dietary and biochemical data are presented in Table 1.

VDBP single nucleotide polymorphisms (SNPs) and the genotype percentage distributions of mothers and neonates are presented in Table 2.

### 3.1. Distribution of Neonatal and Maternal Vitamin D Status According to VDBP Polymorphisms

Distributions of vitamin D status of maternal–neonatal dyads according to VDBP polymorphisms are presented in Table 3, Table 4, Table 5 and Table 6. Data and *p* values were adjusted for maternal height (cm), BMI pre-pregnancy (kg/m^2^), BMI terminal (kg/m^2^), UVB and weeks of gestation by one-way analysis of covariance (ANCOVA). Covariates appearing in the model are evaluated at the following values: BMI pre-pregnancy (kg/m^2^) = 25.09, BMI terminal (kg/m^2^) = 29.73 and weeks of gestation = 38.81. No significant difference was observed in any comparisons after adjustment. Mean concentrations of maternal and neonatal vitamin D status (total 25(OH)D), according to maternal and neonatal VDBP polymorphisms, as well as distribution of different states of maternal and neonatal vitamin D equilibrium, were not different among different genotype profiles of VDBP.

### 3.2. Neonatal Cut-Offs at Birth (≥50 nmol/L vs. ≤50 nmol/L and ≥25 vs. ≤25 nmol/L), According to Neonatal VDBP Polymorphisms

Genotype distribution of neonatal VDBP polymorphisms, using different neonatal cut-offs for 25(OH)D at birth, revealed that no significant differences were evident regarding neonatal vitamin D cut-offs of 25 and 50 nmol/L (Table 7).

### 3.3. Maternal Vitamin D Status at Birth (Cut-Offs at Birth ≤25 vs. ≥25 nmol/L, ≤50 vs. ≥50 nmol/L and ≥75 nmol/L vs. ≤75 nmol/L) According to Maternal VDBP Polymorphisms

By comparing the distributions of maternal VDBP polymorphisms and maternal 25(OH)D concentrations with cut-offs at birth, we revealed that mothers with CC genotype for rs2298850 and CC genotype for rs4588 tended to demonstrate higher 25(OH)D (≥75 nmol/L) during delivery (*p* = 0.05 and *p* = 0.04, respectively), as viewed in Table 8.

### 3.4. Neonatal Vitamin D Status at Birth, According to Maternal VDBP Polymorphisms

There were no significant differences between neonatal 25(OH)D concentrations, with respect to maternal VDBP genotype distribution, using cut-offs of 25 and 50 nmol/L at birth (Table 9 and Table 10).

## 4. Discussion

Apart from its well-established role as the major plasma carrier protein of vitamin D and its metabolites, VDBP is also considered a critical bioregulator of vitamin D equilibrium during pregnancy [16,17]. Fluctuations of VDBP concentrations during pregnancy, resulting from adaptive changes on the maternal–neonatal interface, have been reported to exert significant effects on the vitamin D profile [16,17,18]. However, the effects of the specific genetic profile of VDBP polymorphisms on maternal–neonatal vitamin D status, based on widely adopted 25(OH)D cut-offs at term, have not been investigated previously in the region of southern Europe. Our results revealed the following:

(i) maternal VDBP polymorphisms do not affect neonatal vitamin D concentrations at birth, in any given internationally adopted maternal or neonatal cut-off for 25(OH)D concentrations;
(ii) neonatal VDBP polymorphisms are not implicated in the regulation of neonatal vitamin D status at birth;
(iii) in a maternal cohort not affected by vitamin D supplementation during pregnancy, mothers with CC genotype for rs2298850 and CC genotype for rs4588 tended to demonstrate higher 25(OH)D (≥75 nmol/L) concentrations, after adjustments for biofactors that affect vitamin D equilibrium, including UVB, BMI and weeks of gestation. The fact that this finding was evident in a small cohort implies that a biologically plausible basis, which could explain the profound differences of maternal vitamin D status, was observed in our region [25,26], as well as adding to existing genetic influences on maternal hypovitaminosis D during pregnancy [31].

Available studies regarding the interplay of VDBP polymorphisms with 25(OH)D concentrations are conflicting. In specific, rs12512631 and rs7041 were found to affect maternal and cord-blood concentrations of 25(OH)D [32,33]. Insufficient 25(OH)D concentrations were reported in infants of mothers carrying the rs12512631 “C” allele [30].

In addition, GC rs2282679 polymorphism was associated with achieved 25(OH)D concentrations after cholecalciferol supplementation and during pregnancy [32]. The minor allele for rs7041 was also associated with higher 25(OH)D and rs4588 was associated with lower 25(OH)D levels during pregnancy [34], whereas Chinese pregnant women, with VDBP Gc-1f and Gc-1s genotypes, manifested higher plasma 25(OH)D status compared to women with Gc-2 [35].

VDBP concentrations manifest a variable longitudinal increase during pregnancy [18,36], observed only in women with rs7041 GG or GT genotypes [35,37]. Genetic variations of VDBP polymorphisms could partly explain different supplementation responses during pregnancy, regarding clinical outcomes [6,25,26]. Of major interest in a recent cohort with 815 Chinese women, the influence of variants of rs17467825, rs4588, rs2282679 and rs2298850 on maternal 25(OH)D has been reported to be modified by vitamin D supplementation and sunshine exposure [38]. It is interesting to note that significantly higher levels of serum 25(OH)D in homozygous major allele carriers for the rs2298850 of GC gene were also observed in Parkinson’s disease cases with slower progression [39]. It becomes evident that a country-specific clinical approach and a tailored approach, according to specific lifestyle and genetic profiles of pregnant women, could result in a more pragmatic approach, in terms of vitamin D supplementation and prevention of maternal and neonatal adverse outcomes [40,41].

This study has several limitations. First, the sample size was small and not powered to detect additional differences in other maternal–neonatal cut-offs, but it was sufficiently powered to show significant differences regarding the main aim of the study. Second, the cross-sectional design of the study could not prove a causal relationship. Third, all women were Caucasian, so our results cannot be safely generalized to other ethnicities. On the other hand, the inclusion of both maternal and neonatal polymorphisms, as well as assessment of different cut-offs, could provide a realistic overview of maternal–neonatal dynamics, which is absent in most previous studies of similar design.

In conclusion, this study, from southern Europe, indicates that maternal and neonatal VDBP polymorphisms do not affect neonatal vitamin D status at birth, whereas mothers with CC genotype for rs2298850 and CC genotype for rs4588 demonstrate higher 25(OH)D concentrations. Future larger studies are required to establish a causative effect of these specific polymorphisms, in the attainment of an adequate (≥75 nmol/L) maternal vitamin D status during pregnancy.

## Figures and Tables

**Table 1:** Maternal and neonatal demographic and anthropometric characteristics.

Maternal	
Number (*n*)	66
Age (years)	31.92 ± 6.08
Height (cm)	164.85 ± 5.47
Weight; pre-pregnancy (kg)	67.56 ± 14.54
Weight; term (kg)	85.43 ± 14.30
BMI; pre-pregnancy (kg/m^2^)	24.91 ± 4.81
BMI; term (kg/m^2^)	29.62 ± 5.80
Weeks of gestation (*n*)	38.80 ± 1.56
Smoking [*n* (%)]	10 (0.14)
Alcohol consumption [*n* (%)]	8 (0.11)
Previous live births [*n* (%)]	26 (0.37)
Daily calcium supplementation [*n* (%)]	37 (0.56)
Daily calcium supplementation (mg)	423.07 ± 319.07
Daily dietary calcium intake during 3rd trimester (mg)	792.5 ± 334.0
Daily dietary vitamin D intake during 3rd trimester (mcg)	2.9 ± 1.2
UVB	0.2 ± 0.1
Paternal height (cm)	177.85 ± 6.14
**Neonatal**	
Number (*n*)	66
Gender; Males [*n* (%)]	38 (0.58)
Height (cm)	50.48 ± 1.96
Weight (g)	3292.12 ± 414.25

**Table 2:** Vitamin D binding protein single nucleotide polymorphisms genotype distributions of mothers and neonates (%).

SNP	rs2298850			rs4588			rs7041		
Genotype	CC	CG	GG	CC	CA	AA	GG	GT	TT
Maternal (*n*:%)	33 (0.47)	32 (0.46)	5 (0.07)	32 (0.46)	31 (0.44)	7 (0.10)	19 (0.27)	39 (0.56)	12 (0.17)
Neonatal (*n*:%)	35 (0.50)	28 (0.40)	7 (0.10)	33 (0.47)	30 (0.43)	7 (0.10)	18 (0.26)	38 (0.54)	14 (0.20)

**Table 3:** Mean concentrations of maternal and neonatal 25(OH)D according to VDBP polymorphisms.

Polymorphism	Maternal Genotype	*n*	Maternal 25OHD Level (nmol/L) Mean ± SD	*p* Value	Neonatal Genotype	*n*	Neonatal 25OHD Level (nmol/L) Mean ± SD	*p* Value
rs2298850	CC	33	54.14 ± 30.6	0.96	CC	35	48.84 ± 31.4	0.70
	CG	32	51.35 ± 75.1		CG	28	58.90 ± 78.8	
	GG	5	47.10 ± 5.9		GG	7	43.83 ± 18.4	
rs4588	CC	32	55.26 ± 30.4	0.92	CC	33	50.86 ± 31.5	0.77
	CA	31	50.06 ± 76.3		CA	30	56.75 ± 76.5	
	AA	7	49.14 ± 15.7		AA	7	40.66 ± 19.2	
rs7041	GG	19	55.24 ± 34.2	0.67	GG	18	49.12 ± 30.8	0.63
	GT	39	54.97 ± 68.5		GT	38	57.75 ± 69.9	
	TT	12	39.32 ± 18.6		TT	14	41.91 ± 20.2	

**Table 4:** Distribution of neonatal vitamin D status according to neonatal VDBP genotype polymorphisms.

Polymorphism	Genotype	Deficient *n* = 26 (37%)	Insufficient *n* = 29 (41.5%)	Sufficient *n* = 15 (21.5%)	*p* Value
rs2298850	CC	14 (54%)	11 (38%)	10 (67%)	0.26
	CG	10 (38%)	13 (45%)	5 (33%)	
	GG	2 (8%)	5 (17%)	0 (0%)	
rs4588	CC	13 (50%)	10 (34%)	10 (67%)	0.27
	CA	10 (39%)	15 (52%)	5 (33%)	
	AA	3 (11%)	4 (14%)	0 (0%)	
rs7041	GG	7 (27%)	8 (28%)	3 (20%)	0.42
	GT	14 (54%)	13 (44%)	11 (73%)	
	TT	5 (19%)	8 (28%)	1 (7%)	

**Table 5:** Distribution of neonatal vitamin D status according to maternal VDBP genotype polymorphisms.

Polymorphism	Genotype	Deficient *n* = 26 (37%)	Insufficient *n* = 29 (41.5%)	Sufficient *n* = 15 (21.5%)	*p* Value
rs2298850	CC	13 (50%)	11 (38%)	9 (60%)	0.25
	CG	13 (50%)	14 (48%)	5 (33%)	
	GG	0 (0%)	4 (14%)	1 (7%)	
rs4588	CC	12 (46%)	11 (38%)	9 (60%)	0.09
	CA	14 (54%)	12 (41%)	5 (33%)	
	AA	0 (0%)	6 (21%)	1 (7%)	
rs7041	GG	8 (31%)	6 (21%)	5 (33%)	0.87
	GT	14 (54%)	17 (58%)	8 (54%)	
	TT	4 (15%)	6 (21%)	2 (13%)	

**Table 6:** Distribution of maternal vitamin D status according to maternal VDBP polymorphisms.

Polymorphism	Genotype	Deficient *n* = 18 (26%)	Insufficient *n* = 27 (39%)	Sufficient *n* = 25 (35%)	*p* Value
rs2298850	CC	8 (44%)	10 (37%)	15 (60%)	0.28
	CG	10 (56%)	14 (52%)	8 (32%)	
	GG	0 (0%)	3 (11%)	2 (8%)	
rs4588	CC	7 (39%)	10 (37%)	15 (60%)	0.14
	CA	11 (61%)	13 (48%)	7 (28%)	
	AA	0 (0%)	4 (15%)	3 (12%)	
rs7041	GG	5 (28%)	6 (22%)	8 (32%)	0.87
	GT	10 (56%)	15 (56%)	14 (56%)	
	TT	3 (17%)	6 (22%)	3 (12%)	

**Table 7:** Neonatal vitamin D status at birth (cut-offs at birth ≤25 vs. ≥25 nmol/L and ≤50 vs. ≥50 nmol/L) according to neonatal VDBP polymorphisms.

Polymorphism	Genotype	≤50 nmol/L *n* = 55 (79%)	≥50 nmol/L *n* = 15 (21%)	*p* Value	≤25 nmol/L *n* = 26 (37%)	≥25 nmol/L *n* = 44 (63%)	*p* Value
rs2298850	CC	25 (45%)	10 (67%)	0.20	14 (54%)	21 (48%)	0.83
	CG	23 (42%)	5 (33%)		10 (38%)	18 (41%)	
	GG	7 (13%)	0 (0%)		2 (8%)	5 (11%)	
rs4588	CC	23 (42%)	10 (67%)	0.15	13 (50%)	20 (45.5%)	0.83
	CA	25 (45%)	5 (33%)		10 (39%)	20 (45.5%)	
	AA	7 (13%)	0 (0%)		3 (11%)	4 (9%)	
rs7041	GG	15 (27%)	3 (20%)	0.20	7 (27%)	11 (25%)	0.98
	GT	27 (49%)	11 (73%)		14 (54%)	24 (55%)	
	TT	13 (24%)	1 (7%)		5 (19%)	9 (20%)	

**Table 8:** Maternal vitamin D status at birth (cut-offs at birth ≤25 vs. ≥25 nmol/L, ≤50 vs. ≥50 nmol/L and ≥75 nmol/L vs. ≤75 nmol/L) according to maternal VDBP polymorphisms.

Polymorphism	Genotype	≤25 nmol/L *n* = 18 (26%)	≥25 nmol/L *n* = 52 (74%)	*p* Value	≤50 nmol/L *n* = 44 (63%)	≥50 nmol/L *n* = 26 (37%)	*p* Value	≤75 nmol/L *n* = 57 (37%)	≥75 nmol/L *n* = 13 (63%)	*p* Value
rs2298850	CC	8 (44%)	25 (48%)	0.32	18 (41%)	15 (58%)	0.35	23 (40%)	10 (77%)	0.05
	CG	10 (56%)	22 (42%)		23 (52%)	9 (34%)		29 (51%)	3 (23%)	
	GG	0 (0%)	5 (10%)		3 (7%)	2 (8%)		5 (9%)	0 (0%)	
rs4588	CC	7 (39%)	25 (48%)	0.12	17 (39%)	15 (58%)	0.21	22 (39%)	10 (77%)	0.04
	CA	11 (61%)	20 (39%)		23 (52%)	8 (31%)		29 (51%)	2 (15%)	
	AA	0 (0%)	7 (13%)		4 (9%)	3 (11%)		6 (10%)	1 (8%)	
rs7041	GG	5 (27%)	14 (27%)	0.99	11 (25%)	8 (31%)	0.61	13 (23%)	6 (46%)	0.20
	GT	10 (56%)	29 (56%)		24 (55%)	15 (58%)		33 (58%)	6 (46%)	
	TT	3 (17%)	9 (17%)		9 (20%)	3 (11%)		11 (19%)	1 (8%)	

**Table 9:** Neonatal vitamin D status (25(OH)D ≤25 nmol/L vs. 25(OH)D ≥25 nmol/L) according to genotype distribution of maternal VDBP polymorphisms.

Polymorphism	Genotype	Neonatal Vitamin D Status		*p* Value
		25(OH)D ≤ 25 nmol/L *n* = 44 (63%)	25(OH)D ≥ 25 nmol/L *n* = 26 (37%)	
rs2298850	CC	13 (50%)	20 (46%)	0.20
	CG	13 (50%)	19 (43%)	
	GG	0 (0%)	5 (11%)	
rs4588	CC	12 (46%)	20 (46%)	0.08
	CA	14 (54%)	17 (39%)	
	AA	0 (0%)	7 (16%)	
rs7041	GG	8 (31%)	11 (25%)	0.86
	GT	14 (54%)	25 (57%)	
	TT	4 (15%)	8 (138%)	

**Table 10:** Genotype distribution of maternal VDBP polymorphisms according to neonatal vitamin D status (25(OH)D ≤50 nmol/L vs. 25(OH)D ≥ 50 nmol/L).

Polymorphism	Genotype	Neonatal Vitamin D Status		*p* Value
		25(OH)D ≤50 nmol/L *n* = 55 (79%)	25(OH)D ≥ 50 nmol/L *n* = 15 (21%)	
rs2298850	CC	24 (44%)	9 (60%)	0.52
	CG	27 (49%)	5 (33%)	
	GG	4 (7%)	1 (7%)	
rs4588	CC	23 (42%)	9 (60%)	0.45
	CA	26 (47%)	5 (33%)	
	AA	6 (11%)	1 (7%)	
rs7041	GG	14 (26%)	5 (33%)	0.80
	GT	31 (56%)	8 (54%)	
	TT	10 (18%)	2 (13%)	

## Data Availability

The data presented in this study are available on request from the corresponding author.
